# Identification of Tomato Disease Types and Detection of Infected Areas Based on Deep Convolutional Neural Networks and Object Detection Techniques

**DOI:** 10.1155/2019/9142753

**Published:** 2019-12-16

**Authors:** Qimei Wang, Feng Qi, Minghe Sun, Jianhua Qu, Jie Xue

**Affiliations:** ^1^Business School, Shandong Normal University, Jinan, Shandong, China; ^2^University of Texas at San Antonio, San Antonio, Texas, USA; ^3^Business School, Shandong Normal University, Jinan, Shandong, China

## Abstract

This study develops tomato disease detection methods based on deep convolutional neural networks and object detection models. Two different models, Faster R-CNN and Mask R-CNN, are used in these methods, where Faster R-CNN is used to identify the types of tomato diseases and Mask R-CNN is used to detect and segment the locations and shapes of the infected areas. To select the model that best fits the tomato disease detection task, four different deep convolutional neural networks are combined with the two object detection models. Data are collected from the Internet and the dataset is divided into a training set, a validation set, and a test set used in the experiments. The experimental results show that the proposed models can accurately and quickly identify the eleven tomato disease types and segment the locations and shapes of the infected areas.

## 1. Introduction

Plant diseases have always been a thorny problem in agricultural production and one of the main factors restricting the sustainable development of agriculture. As a common vegetable and important cash crop in China, tomato is widely cultivated in various regions, covering an area of nearly 700 million square meters nationwide. Affected by various factors of the environment, tomato diseases occur frequently. According to the current statistical data, there are as many as 20 types of tomato diseases, which have seriously affected the yield and quality of tomatoes and caused huge economic losses. Therefore, the prevention and treatment of tomato diseases play extremely important roles in tomato production. In the past, disease diagnosis mainly uses artificial recognition methods, including

1. Subjectively judge disease types based on years of planting experience of farmers or consult books on agricultural knowledge
2. Obtain disease specimen pictures and search on the Internet for judgment
3. Consult experts to undertake an analysis of the disease symptoms

Therefore, it is possible for a person with strong professional knowledge to accurately diagnose plant diseases. In general, farmers are not highly educated and do not have the necessary professional knowledge. They usually have a high misjudgment rate of plant diseases and, therefore, are difficult to meet the production requirements of the modern agriculture.

In recent years, the development of computer vision has provided a new way for the accurate diagnosis of tomato diseases. Object detection is an important subject in the field of computer vision. The main task of object detection is to precisely locate the region of interest in the image and determine the specific category of each object. In this study, object detection models and different deep convolutional neural networks (DCNNs) [1] are combined, which can not only identify the types of tomato diseases, but also locate the diseased spots so as to use the appropriate treatment.

**Figure 1:**
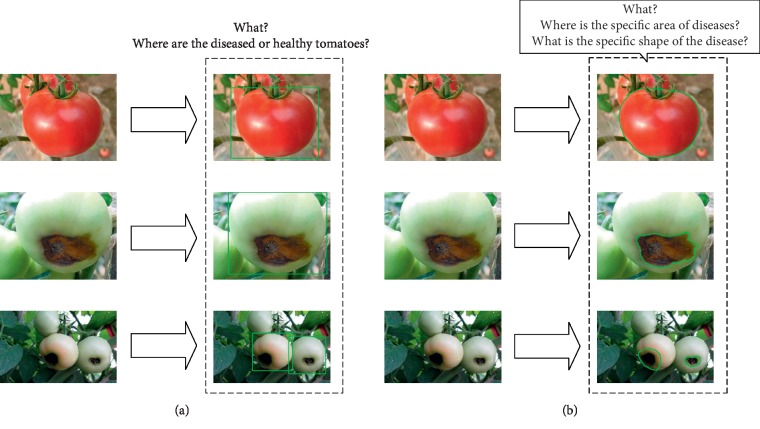
(a) Faster R-CNN, identifying tomato types (healthy vs diseased) and locating the tomatoes. (b) Mask R-CNN, identifying the types of tomato diseases and accurately obtain the location and shape of the lesion.

Two object detection architectures, i.e., Faster R-CNN [2] and Mask R-CNN [3], are combined with four different deep convolutional neural networks. Deep convolutional neural networks are used to automatically extract original image features, and object detection architectures are used to identify, classify, and locate diseased sites in feature maps. The two object detection architectures are used for different purposes. The purpose of Faster R-CNN is to identify and locate diseased tomatoes, while that of Mask R-CNN is to segment specific lesion areas on diseased tomatoes (see Figure 1). Python scripts are used to mark the object and visually show the different purposes of the two architectures.

## 2. Related Works

As early as the 1990s, some scholars applied information technology to plant disease diagnosis and identification. In 1999, Sasakoi et al. [4] used genetic algorithms to establish identification parameters based on spectral reflection characteristics and shape characteristics so as to identify the disease. However, due to insufficient utilization of color and texture information of the diseases, the identification effect was not as effective as expected. In 2002, Bodria et al. [5] used 200 W (radiation wavelength: 360 nm∼430 nm) hernia light source to collect images in a single band and to conduct multispectral identification for wheat infected by different fungi in four bands (radiation wavelengths: 450 nm, 550 nm, 690 nm, and 740 nm).

In 2004, El-Helly et al. [6] used neural networks to identify cucumber powdery mildew, downy mildew, and leaves damaged by leaf miner, with significant effects. In 2005, Liu et al. [7] used back propagation neural networks to predict the occurrences of diseases and insect pests of apple trees using data of the previous 11 years. In 2007, Sammany and Medhat [8] used genetic algorithms to optimize the structure and parameters of neural networks and then used support vector machines and neural networks to identify plant diseases. In 2008, Tellaeche et al. [9] identified weeds between rows of crops in the field by using Hough transform and Gabor filtering based on the perspective of geometry principle of the scene and solved the weed identification problem under different perspectives and different spatial frequencies.

In 2011, Gulhane and Gurjar [10] extracted the color and shape features of the disease spots and combined the original feature map with a feedforward artificial neural network to identify cotton leaf diseases. In 2013, Landge et al. [11] realized automatic detection and recognition of plant diseases based on color, texture, and shape using feature image processing methods and neural networks. Sanyal and Patel [12] used neural networks to identify rice blast, flax spot, and normal leaves, with an identification rate of 89.26%. In 2014, Revathi and Hemalatha [13] extracted edge, CYMK color feature, GA feature, color, texture, and other features by the oblique divergence method and used support vector machines and feedforward artificial neural networks for classification.

In 2016, Mohanty et al. [14] tested 14 crops and 26 diseases in 38 categories in the PlantVillage dataset using the AlexNet and GoogoLeNet networks, respectively, with a maximum identification rate of 99.35%. In 2017, Ramcharan et al. [15] used the Inception v3 network to identify 3 diseases and 2 insect pests of cassava. The recognition rates of brown leaf spot, red mite damage, green mite damage, cassava brown streak disease, and cassava mosaic disease were 98%, 96%, 95%, 98%, and 95%, respectively. In 2018, Ma et al. [16] proposed a deep convolutional neural network to conduct symptom-wise recognition of 4 cucumber diseases with a recognition rate of up to 93.4%. In 2019, Geetharamani and Arun Pandian [17] proposed a nine-layer convolutional neural network to identify 39 kinds of leaves, with an average recognition rate of 96.64%.

Previous works show that deep convolutional neural networks perform well in plant disease recognition. However, if tomato images contain multiple diseases, deep convolutional neural networks can only identify the types of diseases, but cannot obtain the locations of diseased sites and cannot correlate the diseased sites and the disease types. In this study, two object detection architectures combined with deep convolutional neural networks are used to address these issues. These approaches can not only accurately determine the species of tomato diseases, but also obtain the locations of diseased tomatoes and the shapes of the infected areas.

## 3. Methods

This section describes the methods, including the structures and the detection process, used in this study. The Faster R-CNN is used to identify species of tomato diseases and locate the diseased tomatoes. The Mask R-CNN is used to accurately segment the shapes of the infected areas of the diseased tomatoes.

### 3.1. Overall Steps

**Figure 2:**
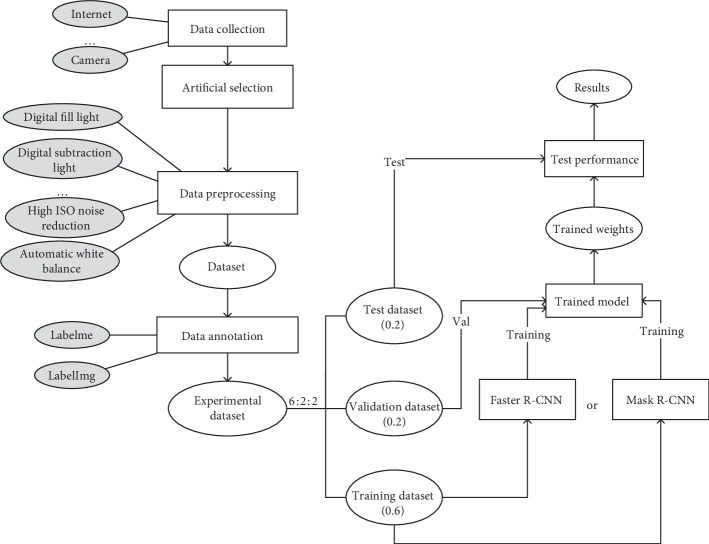
Flowchart for the detection of tomato diseases.

The purpose of this work is to construct two models using deep convolutional neural networks and object detection architectures to identify diseased tomatoes (see Figure 2). Figure 2 is a flowchart of the detection process of tomato diseases. Each part in this figure will be discussed in detail in the following sections.

### 3.2. Deep Convolutional Neural Networks

In order to obtain high recognition rates of tomato diseases, feature extraction neural networks must accurately extract characteristics of the images containing the diseased tomatoes. In previous studies, some typical artificial design features have achieved good performances, such as SIFT (Scale-Invariant Feature Transform) [18] and HOG (Histogram of Oriented Gradient) [19], among others. However, these artificial design features do not have good generalization abilities. As a deep learning model [20], convolutional neural networks have the ability of hierarchical learning [21]. Previous studies [22, 23] show that features obtained through convolutional neural networks have stronger discrimination and generalization abilities than those obtained by artificial designs. In this study, four types of deep convolutional neural networks, VGG-16 [24], ResNet-50 [25], ResNet-101 [25], and MobileNet [26], are used to extract image features (see Table 1).

### 3.3. Tomato Disease Recognition Models Based on Object Detection Architectures

The original R-CNN [27] model uses selective search [28] to obtain candidate region proposals. The sizes of the region proposals are then normalized. AlexNet [29] was used to obtain features in region proposals. Finally, multiple SVMs [30] are used for classification and linear regression for fine-tuning the coordinates of the normalized region proposals. However, the R-CNN [27] does feature extraction for each candidate region proposal separately and repeats for the multiple region proposals, resulting in a large number of repeated calculations. In addition, the normalization of candidate region proposals affects the quality of the final results. To address these issues, the Fast R-CNN [31] uses a RoI layer based on the pyramid principle to unify feature vector sizes of different candidate region proposals. Through multitask learning, the training process is transformed into a single stage so that a large number of reading and writing operations are no longer required. By using the mapping relation to obtain features of different candidate region proposals, the Fast R-CNN only needs to calculate on the whole original image once so as to avoid a large number of repeated calculations. However, both the R-CNN [27] and Fast R-CNN [31] use selective search [28] or edge box detection algorithms [32] based on low-level features to generate region proposals, which is time consuming and produces low-quality candidate regions. Region Proposal Networks (RPNs) [2] based on depth features in the Faster R-CNN proposed in 2017 replaced selective search or edge box detection algorithms. The Mask R-CNN [3] added the function of object segmentation on the basis of the Faster R-CNN so that locations and shapes of object instance could be obtained accurately.

**Figure 3:**
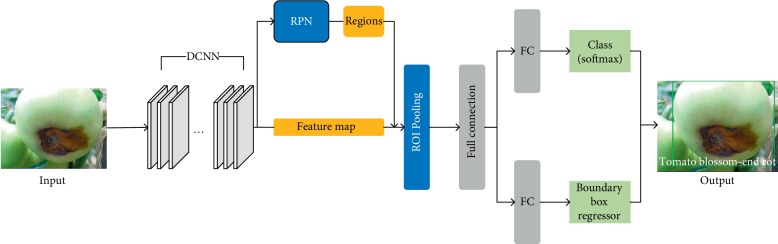
The structure of the Faster R-CNN.

**Figure 4:**
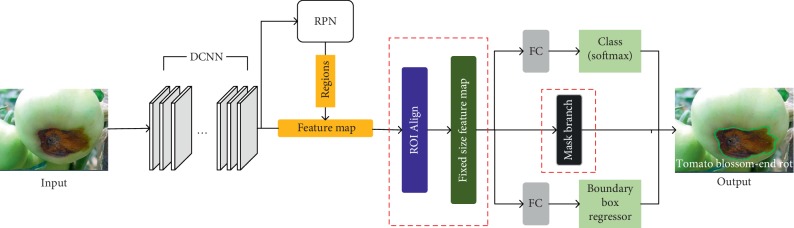
The structure of the Mask R-CNN.

The Faster R-CNN (see Figure 3) and Mask R-CNN (see Figure 4) are two newest approaches used to identify tomato diseases.

In the Faster R-CNN, as shown in Figure 3, the DCNN is used to extract feature maps of the input images, which are used subsequently in the RPN layer and the fully connected layer.

A RPN is mainly used to generate region proposals. In the RPN, in order to generate region proposals, a small network slides on the feature map, which is the output of the deep convolutional neural network in the first step. This small network slides in the feature map and each sliding window is mapped to a low-dimensional feature vector (e.g., VGG-16 has 512 dimensions, followed by a ReLU activation function [33]). This low-dimensional feature vector is the input to the two-collateral fully connected layers, in which the box-regression layer (reg) outputs locations of region proposals and the box-classification layer (cls) judges whether there is a target object in the box. The box is the region proposals mentioned above. On the other hand, the generation of region proposals is realized through a *n* × *n* convolution filter (a *n* × *n* small window slides on the feature map, which is equivalent to a *n* × *n* convolution filter checking the feature map).

The following reg layer and cls layer are also realized through a 1 × 1 convolution filter. The number of region proposals is represented by *k*. Therefore, there are 4*k* output values for the reg layer to encode the coordinates of the *k* region proposals, and 2*k* scores for the cls layer to represent the possibility for each region proposal to contain an object or not. The *k* proposals are parameterized relative to *k* reference boxes called anchors. An anchor is centered at the sliding window and is associated with a scale and aspect ratio. By default 3 scales and 3 aspect ratios are used producing *k*=9 anchors at each sliding window.

The above operations produce a large number of region proposals. Because a sliding window corresponds to 9 anchors, the region proposals of the same object may overlap. The Nonmaximal Suppression (NMS) [34] algorithm is adopted to address this issue. In a nutshell, the IoU (Intersection over Union) [35] between two boxes is computed first, and the box with a low score is discarded if the IoU is greater than a preset threshold. Finally, the retained frames after NMS processing are sorted according to their scores and the *N* boxes on the top are the final region proposals. The obtained region proposals are then mapped by the RPN to the feature map.

The next step is the Region of Interest (ROI) Pooling. In this layer, according to the size and position of the box generated in the RPN, the ROI is clipped from the feature map and processed into the output of fixed size.

The feature map with a fixed size is the input into the fully connected layer for classification. Meanwhile, the boundary box-regression operation is completed to obtain the exact position of the boxes on the tomatoes.

As shown in Figures 3 and 4, the steps of the Mask R-CNN and Faster R-CNN are roughly the same. First, features are extracted through the DCNN, and then region proposals are generated through the RPN layer. Finally, feature maps with fixed sizes are generated for classification and positioning. The improvement made by the Mask R-CNN is that the ROI Pooling layer is optimized into the ROI Align layer and a Mask branch is added while the final classification and positioning are carried out so that shapes of lesion spots can be accurately segmented on the tomato.

**Figure 5:**
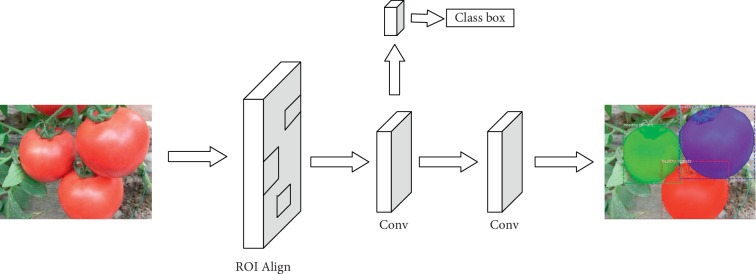
The FCN framework for instance segmentation.

The Faster R-CNN has two main outputs for each ROI. One output is the classification result, i.e., the labels of the boxes, and the other output is the regression result providing the coordinates of the region proposals. The Mask R-CNN adds a third output through the Mask branch, i.e., the Mask R-CNN produces an output for each ROI, which is realized through the FCN (e.g., the two convolution layers in Figure 5).

In the Faster R-CNN, the final inputs of the fully connected layers are required to have a uniform size, but the sizes of ROIs generated by the RPN are not the same. Therefore, an ROI Pooling layer is used to convert ROIs of different sizes to those of the uniform size. The ROIs of the uniform size are the input of the fully connected layers. However, ROI Pooling cannot be applied in the division branch because the corresponding input and output cannot be guaranteed to be the same ROI pixel points. Hence, a new ROI Align layer is proposed to replace the original ROI Pooling layer in the Faster R-CNN. ROI Pooling performs two round quantization operations on ROI coordinates when processing the feature maps and these operations may cause errors. In contrast, ROI Align does not perform quantization while ROI coordinates are always kept as floating points and thus greatly reduces quantization errors.

With these two improvements, the Mask R-CNN can not only determine the types and locations of the lesion spots, but also accurately segment the shapes of the lesion spots.

## 4. Experiments

This section discusses the details of the experiments, including data collection, parameter fitting, and the experimental results. The performance of each model is shown through data and images.

### 4.1. Dataset

#### 4.1.1. Data Collection and Preprocessing

**Figure 6:**
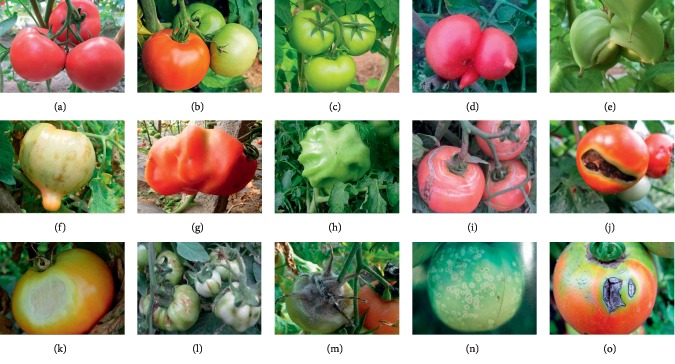
Sample tomato images. (a–c) Healthy tomato. (d and e) Tomato malformed fruit. (f) Tomato blotchy ripening. (g and h) Tomato puffy fruit. (i) Tomato dehiscent fruit. (j) Tomato blossom-end rot. (k) Toamto sunscald. (l) Tomato virus disease. (m) Tomato gray mold. (n) Tomato ulcer disease. (o) Tomato anthracnose.

Pictures from the Internet are taken and screened carefully to ensure correct correspondences between images and disease types. After careful examination, images of ten tomato diseases are sorted out, including tomato malformed fruit, tomato blotchy ripening, tomato puffy fruit, tomato dehiscent fruit, tomato blossom-end rot, tomato sunscald, tomato virus disease, tomato gray mold, tomato ulcer disease, and tomato anthracnose (see Figure 6). Including images of healthy tomatoes, a dataset of eleven types of, with a total of 286, tomato images is used in the experiments. The dataset is divided into a training set, a validation set, and a test set in a ratio of 6 : 2 : 2.

**Figure 7:**
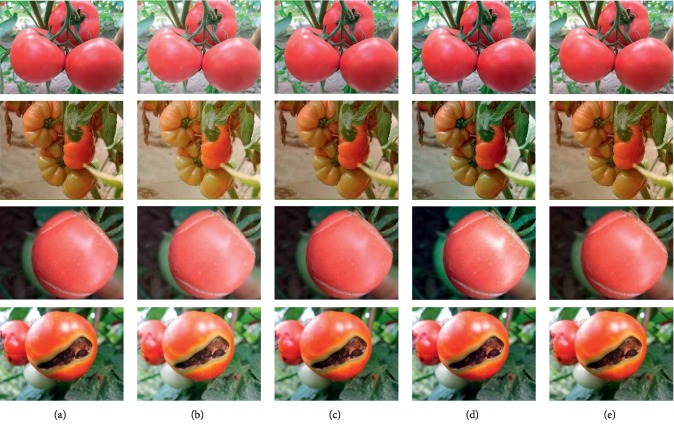
Randomly selected processed images. (a) Original. (b) Digital fill light. (c) Digital subtraction light. (d) Automatic white balance. (e) High ISO noise reduction.

In order to expand the dataset, four methods, i.e., digital fill light, digital subtraction light, automatic white balancing, and high ISO noise reduction, are used for image processing (see Figure 7). Listed from the top to the bottom in Figure 7 are images of healthy tomatoes, tomato malformed fruit, tomato dehiscent fruit, and tomato blossom-end rot. Arranged from left to right, also labeled from *a* to *e*, are the original, digital fill light, digital subtraction light, automatic white balancing, and high ISO noise reduction processed images. The processed dataset contains 1,430 images (see Table 2).

#### 4.1.2. Data Annotation

**Figure 8:**
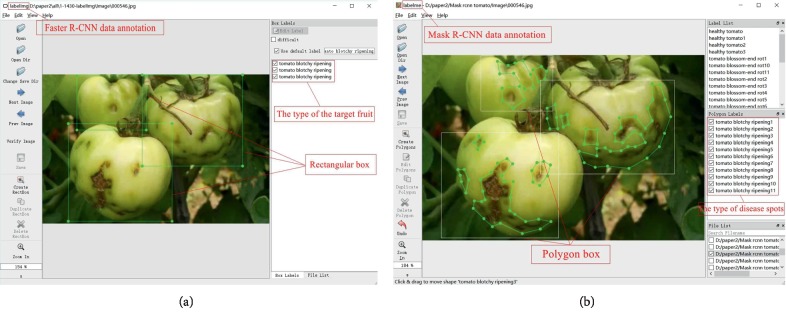
Examples of two data annotation methods. (a) Faster R-CNN data annotation. (b) Mask R-CNN data annotation.

The two different object detection architectures require the images in the training set to be annotated in two different ways (see Figure 8). The Faster R-CNN is mainly used to identify the types and locations of tomato diseases in the images. LabelImg was used to annotate the images used by the Faster R-CNN. The specific operation was to mark the location of a tomato disease spot with a rectangular frame and then annotate it. The Mask R-CNN requires the images to be segmented to obtain more accurate tomato infected areas and spot shapes. Labelme was used to label the images used by the Mask R-CNN. The method was to mark the area and shape of the disease spot with irregular polygons and then label the type of disease spot. Figure 8 gives examples of the two image annotation methods used by the two different object detection architectures.

### 4.2. Experiment Setup

The training set is used to train the model. The validation set is used to give feedbacks about the progress of the training and determine if the training is complete. Finally, the trained model is applied to the test set to evaluate its performance.

The experiments were conducted on a desktop computer with an Intel Core i7 3.70 GHz Processor and an NVidia GeForce GTX 1080 Ti GPU. With the small dataset used in the experiments, the number of iterations of the Faster R-CNN was set to 70,000, and the training results were saved every 5,000 iterations. For the Mask R-CNN, 500 iterations of training are sufficient. In the RPN, boxes with IoU scores larger than a threshold of 0.8 are kept. The remaining parameters are set to the values used in the original works of the Faster/Mask R-CNN reported in the literature [2, 3].

**Figure 9:**
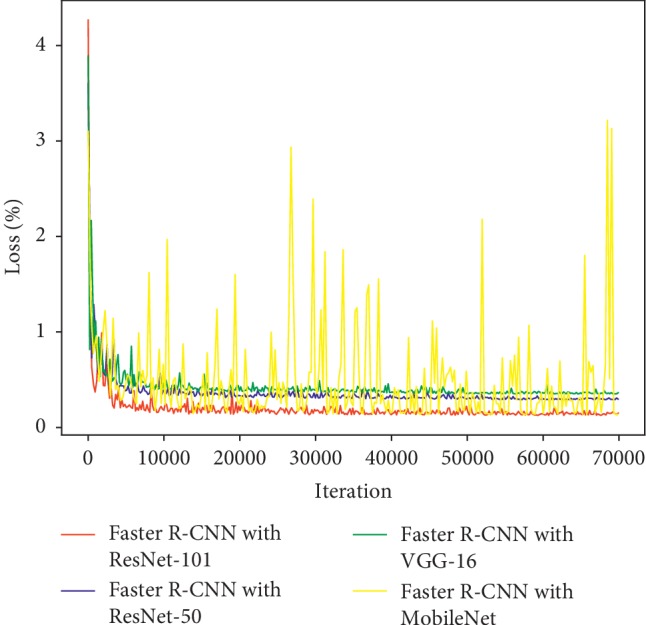
Training loss curves of the Faster R-CNN with different DCNNs.

**Figure 10:**
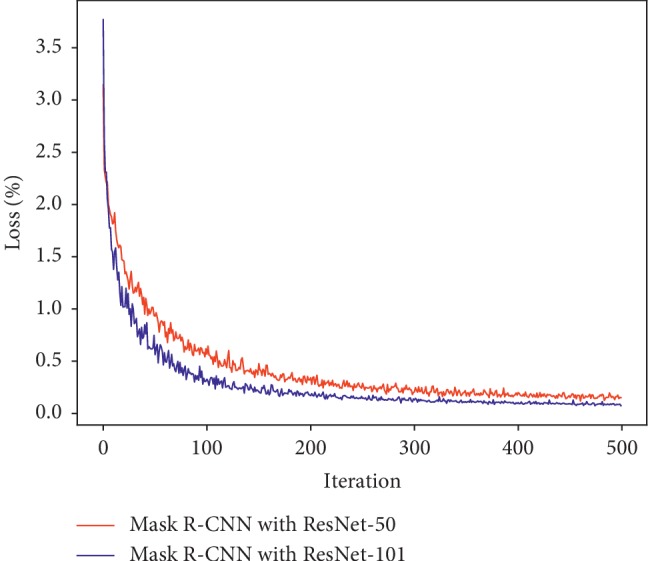
Training loss curves of the Mask R-CNN with different DCNNs.

Figure 9 shows the training loss curves of the Faster R-CNN with 70,000 iterations. The four different models are labeled and coded with different colors in the figure. Except for the loss curve of MobileNet which exhibits a severe shock, the loss curves of the others, including VGG-16, ResNet-50, and ResNet-101, start to stabilize at about 20,000 iterations. In theory, the Faster R-CNN with ResNet-101 has the best performance since it obtains the lowest loss value eventually.  Figure 10 illustrates the loss curves of the Mask R-CNN with 500 iterations. Loss values of both networks started to level off at about 350 iterations. As can be seen, ResNet-101 gets a lower loss value, so that the Mask R-CNN with ResNet-101 performs better than the Mask R-CNN with ResNet-50 in theory.

### 4.3. Quantitative Analysis

Mean Average Precision (mAP), training time, and image detection time were used to measure the performance of each model used in this study.

For a single class in a single image, precision is defined as

(1) $$\begin{array}{c} \text{Precision}=\;\frac{\text{TP}}{\text{TP}+\text{FP}}, \end{array}$$

where  TP(True Positives) represents the number of positive samples correctly classified as positive, i.e., the actual positive samples also correctly classified as positive samples by the classifier, and FP(False Positives) represents the number of negative samples incorrectly classified as positive, i.e., the actual negative samples wrongly classified as positive samples by the classifier.

For multiple images in a single class, Average Precision is defined as

(2) $$\begin{array}{c} \text{Average Precision}=\;\frac{\sum \text{Precision}}{N}, \end{array}$$

where  ∑Precision represents the sum of the precisions for all images and, obviously, *N* is the number of images.

If the dataset includes different classes (like the dataset in this study), a single number is needed to evaluate the performance of the model. The average of Average Precision*s* of all classes, called the mAP, is used for this purpose. For multiple images in multiple classes, the mAP is defined as

(3) $$\begin{array}{c} \text{mAP}=\;\frac{\sum \text{Average }\text{Precision}}{N^{\prime }}, \end{array}$$

where ∑Average Precision represents the sum of the *Average* *Precision* values for all images. Obviously, *N*′ is the number of all classes. The Average Precision and mAP values of tomato images obtained by different diseases detection architectures are shown in Table 3.

In the Faster R-CNN, the mAP value of ResNet-101 is the highest, reaching 88.53%. In addition, ResNet-101 performed best in the detection of single tomato disease types. VGG-16 performed well in three types of tomato diseases, ResNet-50 and MobileNet performed well in five types of tomato diseases, while ResNet-101 performed well in seven types of tomato diseases. In the Mask R-CNN model, ResNet-101 also performed well, achieving high detection rates on all types of tomato diseases, with an mAP value as high as 99.64%.

As can be seen from the table, the performance of the Faster R-CNN in tomato ulcer disease and tomato anthracnose is relatively poor, possibly because the training samples of these two diseases are relatively small, resulting from insufficient data acquired from the Internet. In the Mask R-CNN, because the ROI Align layer corrects the learning errors, the detection result is better than that of the Faster R-CNN.

In addition to detection rates, the efficiency of the models is also a crucial criterion (see Tables 4 and 5).  Table 4 presents the training times in hours and Table 5 presents the detection times in seconds, respectively, taken by the six architectures on all the tomato images.

In the Faster R-CNN, with the deepening of convolutional neural networks, the training time is getting longer and longer. The time of ResNet-101 is the longest, reaching 23.25 hours. MobileNet, a lightweight network that can be ported to mobile devices, offers a much shorter training time of just 12.52 hours.

Because the Mask R-CNN has a more complex structure and a larger amount of computation, the training time of the Mask R-CNN is longer than that of the Faster R-CNN for the same deep convolutional neural networks.

As can be seen from the table above, the deeper the convolutional neural network is, the more complex the model structure is, and the longer the detection time is.

### 4.4. Qualitative Analysis

#### 4.4.1. Samples of Faster R-CNN Detection Results

**Figure 11:**
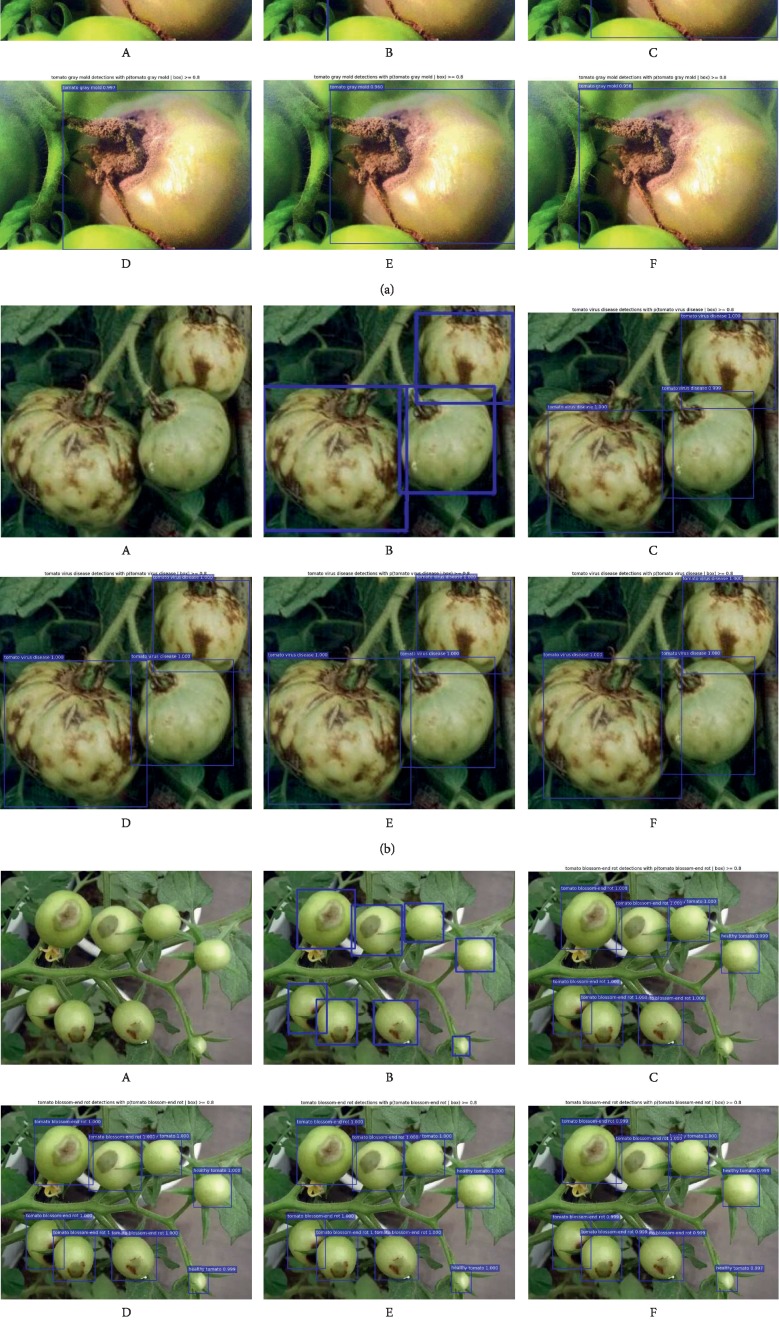
Detection results of the Faster R-CNN in three tomato diseases (a–c) (A) Original; (B) annotated; (C) Faster R-CNN with VGG-16; (D) Faster R-CNN with ResNet-50; (E) Faster R-CNN with ResNet-101; (F) Faster R-CNN with MobileNet.

Figure 11 intuitively shows the detection results of the Faster R-CNN with different DCNNs for three tomato diseases. The original image, the image with annotated boxes, and the detection results are presented for each disease for comparison purpose. As shown in the figure, the model used in this study can effectively detect the types and locations of the tomato diseases.

#### 4.4.2. Samples of Mask R-CNN Detection Results

**Figure 12:**
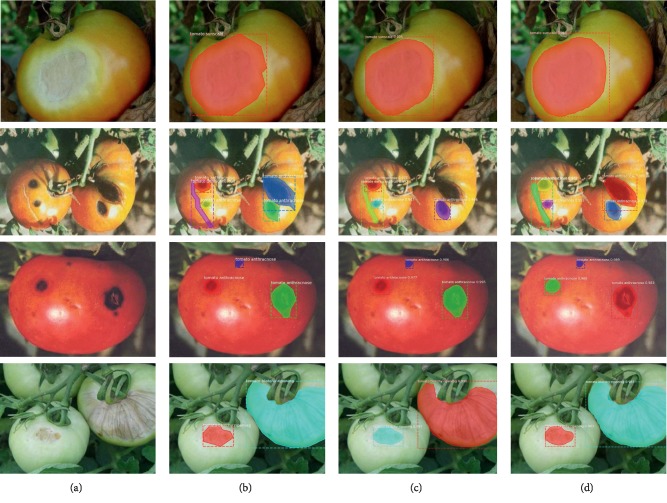
Detection results of the Mask R-CNN. (a) Original; (b) annotated; (c) Mask R-CNN with ResNet-50; (d) Mask R-CNN with ResNet-101.

Figure 12 shows the detection results of the Mask R-CNN with different DCNNs for four tomato diseases. The original image, the image with annotated boxes, and the images of the detection results are presented for each disease for comparison purposes. As shown in the figure, the model used in this study can effectively detect the tomato disease types and accurately segment the shapes of the infected areas.

#### 4.4.3. Imperfect Disease Detection

**Figure 13:**
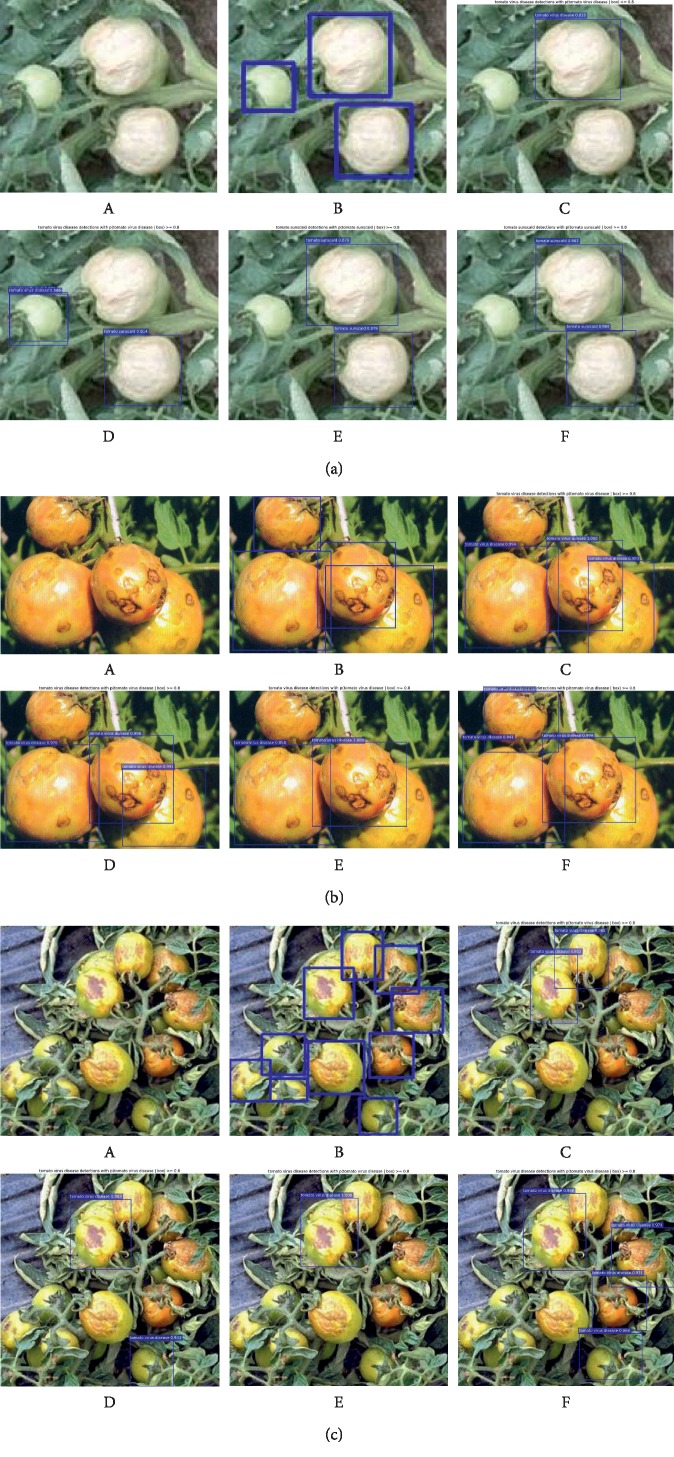
Examples of failed cases of the Faster R-CNN in three tomato diseases (a–c). (A) Original; (B) annotated; (C) Faster R-CNN with VGG-16; (D) Faster R-CNN with ResNet-50; (E) Faster R-CNN with ResNet-101; (F) Faster R-CNN with MobileNet.

**Figure 14:**
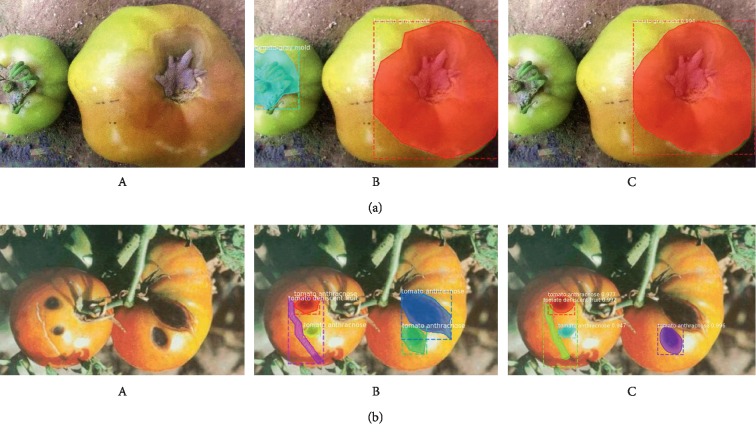
Examples of failed cases of the Mask R-CNN in toamto diseases (a and b). (A) Original; (B) annotated; (C) Mask R-CNN with ResNet-50.

Although the tomato disease detection architectures in this study show superior performance, they also show deficiencies in some aspects. Examples of insufficient detection by the Faster R-CNN are shown in Figure 13 and some others by the Mask R-CNN are shown in Figure 14. As shown in Figures 13 and 14, due to insufficient training images or low image resolutions, the proposed tomato disease detection architectures failed to detect some tomato disease types or infected areas.

## 5. Discussion

In this study, tomato disease detection architectures based on deep convolutional neural networks and object detection models are proposed. Four different neural networks were selected and combined with two object detection models. Experiments with data collected from the Internet show that the proposed methods are very accurate and efficient in detecting tomato disease types and in segmenting shapes of infected areas. Experiment results indicate that ResNet-101 has the highest detection rate, but takes the longest time for training and detection. MobileNet has the shortest detection time, but is less accurate than ResNet-101. In general, different models can be chosen according to the actual needs. Dataset in this study also includes a variety of complex backgrounds, allowing the architectures to improve their abilities to recognize complex images.

There are several future research directions. One direction is to expand the dataset so as to obtain more accurate results. Another direction is to find a way to solve the problem of detection failures caused by low image resolutions. One more direction is to extend this method from tomatoes to other crops.

## Figures and Tables

**Table 1:** Details of the four deep convolutional neural networks.

DCNN	Parameters (M)	Number of layers	Top-1 error (%)^1^	Top-5 error (%)^2^
VGG-16	138	16	28.07	9.33
ResNet-50	25	50	22.85	6.71
ResNet-101	42.6	101	21.75	6.05
MobileNet	3.3	28	—	—

^1^The Top-1 error rate on ImageNet Validation; ^2^the Top-5 error rate on ImageNet Validation; the symbol “-” indicates that no corresponding data are given in the original paper.

**Table 2:** The specific information of the dataset.

Diseases	Original^1^	Expanded^2^	Training^3^	Validation^4^	Test^5^
Healthy tomato	64	320	150	105	65
Tomato malformed fruit	38	190	90	60	40
Tomato blotchy ripening	16	80	35	25	20
Tomato puffy fruit	22	110	50	35	25
Tomato dehiscent fruit	35	175	80	60	35
Tomato blossom-end rot	18	90	40	30	20
Tomato sunscald	14	70	30	25	15
Tomato virus disease	34	170	80	55	35
Tomato gray mold	28	140	65	45	30
Tomato ulcer disease	9	45	20	15	10
Tomato anthracnose	8	40	15	15	10
Total	286	1430	655	470	305

^1^Number of original images; ^2^number of processed images; ^3^number of images in the training set; ^4^number of images in the validation set; ^5^number of images in the test set.

**Table 3:** The Average Precision and the mAP values of the tomato images obtained by different detection architectures.

	Faster R-CNN (%)			Mask R-CNN (%)		
Diseases	VGG-16	ResNet-50	ResNet-101	MobileNet	ResNet-50	ResNet-101
Healthy tomato	90.62	**90.66**	90.54	90.39	**100.00**	**100.00**
Tomato malformed fruit	94.20	99.82	**100.00**	**100.00**	**100.00**	**100.00**
Tomato blotchy ripening	**100.00**	**100.00**	**100.00**	**100.00**	**100.00**	**100.00**
Tomato puffy fruit	70.59	71.30	**73.77**	71.70	**100.00**	**100.00**
Tomato dehiscent fruit	**100.00**	**100.00**	**100.00**	**100.00**	98.88	**100.00**
Tomato blossom-end rot	70.00	97.80	**98.20**	97.50	**100.00**	**100.00**
Tomato sunscald	94.18	89.05	98.33	**100.00**	**100.00**	**100.00**
Tomato virus disease	**77.96**	75.89	73.45	77.44	99.52	**100.00**
Tomato gray mold	90.43	**100.00**	**100.00**	**100.00**	93.33	**100.00**
Tomato ulcer disease	79.80	67.17	**83.47**	67.00	**100.00**	**100.00**
Tomato anthracnose	79.24	**80.86**	53.10	68.26	92.00	**96.00**
mAP	86.09	88.41	**88.53**	88.39	98.52	**99.64**

Bold faces are the detection results of the architecture with the best performance.

**Table 4:** Training time.

Object detection architecture	DCNN	Training time (h)
Faster R-CNN	VGG-16	20.38
	ResNet-50	21.60
	ResNet-101	23.25
	MobileNet	**12.52**

Mask R-CNN	ResNet-50	**22.40**
	ResNet-101	23.82

Bold faces indicate the minimum training time of the model.

**Table 5:** Detection time.

	Detection time^1^ (s)					
	Faster R-CNN			Mask R-CNN		
Diseases	VGG-16	ResNet-50	ResNet-101	MobileNet	ResNet-50	ResNet-101
Healthy tomato	0.158	0.160	0.174	**0.088**	**0.166**	0.186
Tomato malformed fruit	0.164	0.177	0.192	**0.103**	**0.189**	0.200
Tomato blotchy ripening	0.234	0.242	0.268	**0.143**	**0.261**	0.279
Tomato puffy fruit	0.209	0.226	0.243	**0.164**	**0.236**	0.253
Tomato dehiscent fruit	0.165	0.173	0.193	**0.109**	**0.184**	0.204
Tomato blossom-end rot	0.238	0.240	0.264	**0.143**	**0.250**	0.275
Tomato sunscald	0.233	0.262	0.291	**0.161**	**0.282**	0.311
Tomato virus disease	0.205	0.213	0.222	**0.114**	**0.222**	0.232
Tomato gray mold	0.218	0.227	0.231	**0.123**	**0.238**	0.242
Tomato ulcer disease	0.318	0.326	0.365	**0.220**	**0.345**	0.377
Tomato anthracnose	0.411	0.490	0.547	**0.212**	**0.585**	0.667
Mean time	0.202	0.209	0.226	**0.123**	**0.227**	0.246

^1^The detection time of the model on each tomato disease types; bold face indicates the minimum detection time of the model.

## Data Availability

The image data used in this study are available from the first author (474458464@qq.com) upon request.
