# Corrigendum to “Identification of Tomato Disease Types and Detection of Infected Areas Based on Deep Convolutional Neural Networks and Object Detection Techniques”

**DOI:** 10.1155/2021/3751479

**Published:** 2021-02-01

**Authors:** Qimei Wang, Feng Qi, Minghe Sun, Jianhua Qu, Jie Xue

**Affiliations:** ^1^Business School, Shandong Normal University, Jinan, Shandong, China; ^2^University of Texas at San Antonio, San Antonio, TX, USA

In the article titled “Identification of Tomato Disease Types and Detection of Infected Areas Based on Deep Convolutional Neural Networks and Object Detection Techniques” [1], there was an error in the corresponding author affiliation, where the author Feng Qi's affiliation “University of Texas at San Antonio, San Antonio, Texas, USA” should be corrected to “Business School, Shandong Normal University, Jinan, Shandong, China.”

The corrected corresponding author's affiliation is shown in the author information above.
